# Cognitive Outcomes of Children With Sagittal Craniosynostosis Treated With Either Endoscopic or Open Calvarial Vault Surgery

**DOI:** 10.1001/jamanetworkopen.2024.8762

**Published:** 2024-04-29

**Authors:** Suresh N. Magge, Annahita R. Fotouhi, Virginia Allhusen, Brent R. Collett, Gary B. Skolnick, Sybill D. Naidoo, Matthew D. Smyth, Robert F. Keating, Raj Vyas, Gary F. Rogers, Kamlesh B. Patel

**Affiliations:** 1 Department of Neurosurgery, University of Michigan, Ann Arbor; 2 Division of Neurosurgery, Children’s Hospital of Orange County Neuroscience Institute, Children’s Hospital of Orange County, Orange, California; 3 Division of Neurosurgery, Children’s National Hospital, Washington, DC; 4 Division of Plastic and Reconstructive Surgery, Department of Surgery, Washington University School of Medicine, St Louis, Missouri; 5 College of Medicine, University of Kentucky, Lexington; 6 Children’s Hospital of Orange County Research Institute, Orange, California; 7 Center for Child Health, Behavior, and Development, Seattle Children’s Research Institute, Seattle, Washington; 8 Division of Neurosurgery, Johns Hopkins All Children’s Hospital, St Petersburg, Florida; 9 Division of Plastic Surgery, Children’s Hospital of Orange County, Orange, California; 10 Department of Plastic Surgery, University of California Irvine; 11 Division of Plastic and Reconstructive Surgery, Children’s National Hospital, Washington, DC

## Abstract

**Importance:**

Several studies have reported a higher incidence of neurodevelopmental delays and cognitive deficits in patients with single-suture craniosynostosis; however, there are few studies examining the associations of repair type with cognitive outcomes.

**Objective:**

To measure differences in neuropsychological outcomes between school-age children who were treated for sagittal craniosynostosis and unaffected controls and explore differences in cognitive function among children with sagittal craniosynostosis who were previously treated with either endoscopic strip craniectomy or open calvarial vault surgery.

**Design, Setting, and Participants:**

This cohort study was performed between 2018 and 2022. Eligible participants included patients aged 5 to 17 years who had previously been seen as infants or toddlers (<3 years) at 1 of 3 surgical centers for craniosynostosis repair with either endoscopic surgery or open calvarial vault surgery. A separate cohort of unaffected controls were included for comparison. Data analysis was conducted from November 2023 to February 2024.

**Exposures:**

Open calvarial vault surgery or endoscopic repair for single-suture craniosynostosis.

**Main Outcomes and Measures:**

The primary outcome was the Differential Ability Scales-II (DAS-II) General Conceptual Ability (GCA) score, an index for overall intellectual ability. Secondary outcomes included DAS-II subscale scores (Verbal Ability, Nonverbal Reasoning, Spatial Ability, Working Memory, and Processing Speed), and Patient-Reported Outcomes Measurement Information System (PROMIS) cognitive function scores.

**Results:**

A total of 81 patients with sagittal craniosynostosis (59 male [73%]; 22 female [27%]) and 141 controls (81 male [57%]; 60 female [43%]) were included. Of the 81 participants with sagittal craniosynostosis, 46 underwent endoscopic repair and 35 underwent open repair. Median (range) age at time of follow-up assessment was 7.7 (5.0-14.8) years for children with sagittal craniosynostosis and median age at assessment was 8.5 (7.7-10.5) years for controls. After controlling for age at assessment, sex, and socioeconomic status, there was no statistically significant or clinically meaningful difference in GCA scores between children who underwent endoscopic repair (adjusted mean score, 100; 95% CI, 96-104) and open repair (adjusted mean score, 103; 95% CI, 98-108) (*P* > .99). We found no significant difference in PROMIS scores between repair types (median [range] for endoscopic repair 54 [31-68] vs median [range] for open repair 50 [32-63]; *P* = .14). When comparing the treatment groups with the unaffected controls, differences in subscale scores for GCA and working memory were observed but were within normal range.

**Conclusions and Relevance:**

In this cohort study, there were no statistically or clinically significant differences in cognitive outcomes among school-age children by and type of surgical procedure used to repair nonsyndromic sagittal craniosynostosis. These findings suggest primary care clinicians should be educated about different options for craniosynostosis surgery to ensure early referral of these patients so that all treatment options remain viable.

## Key Points

**Question:**

Is there a difference in cognitive outcomes between school-age children who had undergone minimally invasive endoscopic surgery vs children who had undergone open calvarial vault surgery to treat nonsyndromic sagittal craniosynostosis, and if so, how do these differences compare with children who do not have sagittal craniosynostosis?

**Findings:**

In this cohort study of 81 children with sagittal craniosynostosis and 141 controls, there were no statistically or clinically significant differences in outcomes by type of surgical procedure used to repair nonsyndromic sagittal craniosynostosis. When comparing the treatment groups with the unaffected controls, significant differences in scores for general conceptual ability and working memory were observed but were all within normal range.

**Meaning:**

These findings suggest that educating primary care clinicians about different options for craniosynostosis surgery is crucial to ensure early referral of these patients so that all treatment options are still viable.

## Teaser

This cohort study compares long-term cognitive outcomes of school-age children with sagittal craniosynostosis who had undergone either endoscopic surgery or open calvarial vault surgery.

## Introduction

Sagittal craniosynostosis is a condition in which the sagittal suture in the infant skull fuses prematurely. This condition must be treated surgically to allow for the rapid growth and expansion of the brain that occurs in the first 2 years. Traditional management of craniosynostosis involved open cranial surgery, in which the skull is reshaped to allow for normal brain growth. However, over the last 30 years, more centers have been offering minimally invasive endoscopic surgery to treat sagittal craniosynostosis with comparable safety and efficacy.^1,2,3,4^ Benefits of the endoscopic approach include shorter operative times, reduced costs, shorter hospital stays, and fewer blood transfusions.^5^ Endoscopic techniques vary depending on the center but usually involve using small incisions and an endoscope to remove the fused sagittal suture without extensive cranial remodeling.^6,7^ The surgery is typically performed at 2 to 4 months of age.^8^ This age is considered ideal because the head is growing rapidly and the skull is relatively malleable, and thus more conducive to reshaping. For older children, open calvarial vault surgery is typically the preferred approach.^9^ The decision to perform open vs endoscopic surgery is typically based on factors such as patient age, head shape, associated craniofacial anomalies, access to postoperative orthotic therapy, and surgeon preference.^10^

A primary concern when craniosynostosis is present is that the growth of the brain will be slowed, possibly leading to neurocognitive delay.^11^ Multiple studies^12,13,14^ have reported neurodevelopmental delays and cognitive deficits in children previously treated with surgery for single-suture craniosynostosis. However, a recent meta-analysis^15^ of cognitive, behavioral, and psychological outcomes for patients with a history of sagittal craniosynostosis found a wide range of reported outcomes with no clear consensus, although the authors concluded that some patients with sagittal craniosynostosis experience deficits in 1 or more of these domains.

Similar studies of older patients are less common.^12^ Becker et al^16^ evaluated school-age children (mean age, 6 years) and found speech, cognitive, and/or behavioral abnormalities in patients who had undergone traditional open repair. Preoperative morphological severity has not shown to be associated with the incidence of speech-language and psychological concerns.^17^ Another study^18^ reported deficits in intelligence quotient and math computation in patients who had undergone open repair, compared with unaffected controls. Notably, deficits were more severe in patients with unicoronal or metopic craniosynostosis than in those with sagittal craniosynostosis,^18^ a finding that was also reported more recently.^19^ Bellew et al^20^ found that surgery done at younger ages was associated with better neurocognitive outcomes when measured at 10 and 15 years of age.

The association of surgical technique with cognitive outcomes has been debated. One study^21^ of 70 patients showed significantly greater verbal and reading skills in patients treated with traditional whole-vault cranioplasty, compared with those treated using an open strip craniectomy technique (without postoperative orthotics). However, the study’s^21^ methodology and conclusions have been debated, leaving open the question of whether minimally invasive and open repair of nonsyndromic craniosynostosis achieve equivalent cognitive outcomes.^22^ A large-scale study of cognitive outcomes post–endoscopic strip craniectomy remains lacking.^23,24^

The goals of this study were to examine differences in cognitive function between school-age children who were treated for sagittal craniosynostosis and unaffected controls and explore differences in cognitive function between children with sagittal craniosynostosis who were treated with endoscopic surgery vs open cranial vault surgery.

## Methods

This multi-institutional cohort study included St Louis Children’s Hospital (St. Louis, Missouri), Children’s Hospital of Orange County (Orange, California), and Children’s National Hospital (Washington, DC). Recruitment and enrollment were conducted from November 2018 to February 2022. All parents and/or guardians provided written informed consent. This study was approved by the institutional review boards at each participating hospital. The study followed the Strengthening the Reporting of Observational Studies in Epidemiology (STROBE) reporting guideline.

### Participant Selection

**Figure 1.**
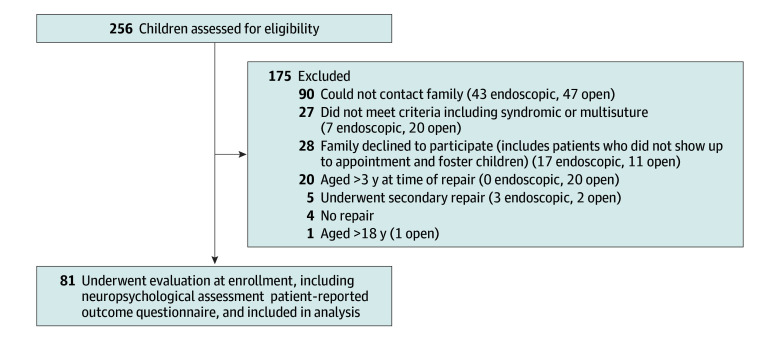
Patient Screening and Inclusion

All patients aged 5 to 17 years who had been treated with primary surgical correction of isolated sagittal craniosynostosis by 3 years of age were eligible for inclusion in the study. Patients with multisuture involvement, any craniofacial syndrome, other medical conditions that could affect neurodevelopment, or secondary repair were excluded. Eligible patients were screened, and their parents or guardians were contacted. Of 256 patients assessed for eligibility, 175 were excluded (see Figure 1 for reasons for exclusion by surgery type). The most common reason for exclusion was that the family could not be contacted because many of the patients were many years from their original surgical repair (90 cases).

### Surgery Types

Patients in the endoscopic group were treated with endoscopic strip craniectomy followed by orthotic helmet therapy. In these patients, an endoscope was used to make a narrow vertex strip craniectomy or wide vertex strip craniectomy with or without barrel staves through 2 small incisions.^6,7,25^ A postoperative helmet was worn until about 10 to 12 months of age. Information on helmet therapy compliance was not systematically collected and could therefore not be considered in this analysis. Patients in the open calvarial vault surgery group were treated using cranial vault reconstruction or modified pi craniectomy based on skull deformity and surgeon preference.^9^

### Unaffected Controls

Deidentified data from a previous longitudinal cohort study at Seattle Children’s Research Institute were used for comparison.^26^ The cohort included healthy controls with positional plagiocephaly followed from infancy through early school-age. Control participants were eligible if they did not have any evidence of craniosynostosis or other craniofacial condition (confirmed with 3-dimensional imaging), any diagnosed neurodevelopmental condition, or severe sensory impairments (eg, deafness or blindness).^27^ Unaffected controls completed cognitive assessment at a mean (range) age of 8 (8-11) years.

### Assessments

Neuropsychological assessment was completed using the Differential Ability Scales, Second Edition (DAS-II).^28^ All tests were administered by qualified psychometrists with previous experience completing standardized testing with young children. During the same visit, parents or guardians completed a semistructured interview to provide information regarding familial demographics and participant involvement in developmental interventions (eg, physical or occupational therapy and speech-language therapy). The Hollingshead Four-Factor Index was used to assess socioeconomic status (SES).^29^ Total scores on the Hollingshead Four-Factor Index range from 8 to 66, with higher scores reflecting higher SES (category V, 8-19; category IV, 20-29; category III, 30-39; category II, 40-54; category I, 55-66). Patient-reported cognitive function was assessed using the Patient-Reported Outcomes Measurement Information System (PROMIS) Cognitive Function Short Form.^30,31^

### Neurodevelopment Outcome Measures

The primary outcome measure was the DAS-II composite outcome for general conceptual ability, an index of conceptual and reasoning abilities. Secondary outcome measures were DAS-II composite scores for verbal ability, nonverbal reasoning, working memory, and processing speed. Primary and secondary outcomes were compared among the endoscopic repair, open repair, and control cohorts.

The DAS-II is a reliable, validated measure of cognitive ability used to identify representative outcomes and sources of variation for children across multiple domains.^26,32,33^ Norm-referenced standard scores (mean [SD], 100 [15]) were generated for all outcomes, with higher scores indicating better performance. Quality control for DAS-II administration and scoring was performed as follows. Select DAS-II record forms were reviewed for quality assurance by an author (B.R.C.; eg, to ensure that ages were calculated correctly, basal and ceiling rules were followed, and scoring was reviewed), including the first administration by each examiner and a randomly selected 10% thereafter. There were no substantive discrepancies or issues found with the DAS-II assessments reviewed.

### Patient Reported Outcome Measure

Patients who underwent endoscopic or open repair for craniosynostosis were given the school-age PROMIS Cognitive Function questionnaire. Parents self-reported certain demographic items such as race and ethnicity. Race categories included African American, Asian, White, multiracial, and unknown race; ethnicity categories included Hispanic or Latino and non–Hispanic or Latino. Race and ethnicity were included for completeness and to allow for subanalysis. The PROMIS Pediatric/Parent Proxy (version 1.0 [short form]) is a validated outcome measure of self-reported cognitive function that has been shown to be useful for evaluating patients diagnosed with craniosynostosis.^31,34^ This 7-item instrument assesses patient-perceived cognitive deficits in domains such as mental acuity, concentration, memory, and fluency. Sample items include, “I forget schoolwork I need to do,” “It’s hard for me to concentrate,” and “I have to work hard to pay attention, or I will make a mistake.” The patient self-report form was used for participants 8 years and older, and a corresponding parent proxy form was used for participants younger than 8 years per PROMIS administration guidelines. Final scores are normalized T-scores (mean [SD], 50 [10]). Scores greater than 55 are deemed within normal limits.^31^ Scores were compared between patients who underwent endoscopic vs open repair. The PROMIS questionnaire was not administered to participants in the control group.

### Statistical Analysis

Categorical variables are described in terms of frequencies and percentages; continuous variables are described using mean and SD or median and range, as appropriate. Normality of continuous variables was assessed using the Shapiro-Wilk test. Differences between study groups were compared with 1-way analysis of variance or Kruskal-Wallis *H* test. SES was analyzed with Fisher exact test. Univariate comparisons between centers (found in eTable 1 and eTable 2 in Supplement 1) used the *t*, 1-way analysis of variance, Mann-Whitney *U*, Kruskal-Wallis *H*, or Fisher exact test, as appropriate. Comparisons of cognitive function per the PROMIS test were assessed with the Mann-Whitney *U* test. Between-group differences in means and 95% CIs were calculated. Descriptive and univariate comparisons of demographics and outcomes between sites were calculated. Analyses of covariance models were calculated to compare the association of treatment (endoscopic repair, open repair, and control) with DAS-II composite outcomes. All analyses were adjusted for age at DAS-II assessment, sex, and SES (ie, Hollingshead total score). The covariates were selected given their known association with measures of cognitive function.^35,36^ Post hoc pairwise comparisons were corrected with the Bonferroni method. For each DAS-II composite outcome score, a between-group difference greater than 5 points was defined as the minimally clinical important difference in the patient’s cognitive outcome.^37,38^ All computed analyses were 2-tailed and values of *P* < .05 were considered significant. Missing data were treated on a casewise basis. For contingency tables larger than 2 × 2, Fisher exact testing was performed using R statistical software version 4.2.1 (R Project for Statistical Computing). All other analysis was performed using SPSS Statistics versions 28 and 29 (IBM). Data analysis occurred from November 2023 to Februrary 2024.

## Results

A total of 222 participants were included in the study, including 81 patients with sagittal craniosynostosis (59 male [73%]; 22 female [27%]) and 141 controls (81 male [57%]; 60 female [43%]). Of the 81 participants with sagittal craniosynostosis who met inclusion criteria and were enrolled in the study, 46 (57%) had undergone endoscopic repair and 35 (43%) had undergone open repair. Of the 118 children with sagittal craniosynostosis who met eligibility criteria but were not enrolled, 60 children had undergone endoscopic repair (51%) and 58 children had undergone open repair (49%). Median (range) age at the time of initial presentation was 1.9 (0.7-5.9) months for the endoscopic repair cohort and 6.7 (0.2-32.1) months for the open repair cohort. Median (range) age at time of follow-up assessment was 7.7 (5.0-14.8) years for children with sagittal craniosynostosis and median age at assessment was 8.5 (7.7-10.5) years for controls. The disproportionate number of males with sagittal craniosynostosis seen in this sample was in line with that reported in the literature for incidence of sagittal craniosynostosis, with estimates of male-female occurrence of 3.5:1.^39^ Notably, the approximate 3:1 male-female ratio was comparable for the open and endoscopic groups in this sample. Demographic characteristics are presented in Table 1. There were no statistically significant differences in the mean Hollingshead total scores among the three cohorts of participants. However, statistically significant differences were present when comparing the distributions of Hollingshead categories. Specifically, the proportion of patients in higher SES categories was lower in the open repair group than in the unaffected controls or the endoscopic repair group. The distributions among SES categories were similar in the control and endoscopic repair group.

**Table 1.** Demographic Characteristics of the Unaffected Controls, Endoscopic Repair, and Open Repair Cohorts

Characteristic	Participants by procedure, No. (%) (N = 222)		
	Control (n = 141)	Endoscopic repair (n = 46)	Open repair (n = 35)
Age at initial presentation, median (range), mo	NA	1.9 (0.7-5.9)	6.7 (0.2-32.1)
Age at Differential Ability Scales-II testing, median (range) y	8.5 (7.7-10.5)	7.0 (5.3-10.6)	8.5 (5.0-14.8)
Sex			
Female	60 (43)	13 (28)	9 (26)
Male	81 (57)	33 (72)	26 (74)
Race^a^			
African American	6 (4)	4 (9)	2 (6)
Asian	4 (3)	0	2 (6)
White	96 (68)	36 (78)	27 (77)
Multiracial	35 (25)	4 (9)	0
Unknown	0	2 (4)	4 (11)
Ethnicity^a^			
Hispanic or Latino	18 (13)	3 (7)	4 (11)
Non–Hispanic or Latino	123 (87)	43 (93)	31 (89)
Gestation, wk			
≥37	NA	43 (94)	32 (91)
<37	NA	3 (6)	3 (9)
Hollingshead Four Factor Index of Social Status total score, mean (SD)	47 (10)	48 (13)	43 (15)
Hollingshead category^b^			
I	36 (26)	18 (39)	10 (29)
II	74 (53)	17 (37)	10 (29)
III	23 (16)	9 (20)	11 (31)
IV	7 (5)	1 (2)	0
V	1 (1)	1 (2)	4 (11)

Abbreviation: NA, not applicable.

^a^ Race and ethnicity were self-reported or reported by a parent or guardian.

^b^ Highest socioeconomic status level is Hollingshead category I.

Participant and familial characteristics of the endoscopic and open repair cohorts are described in Table 2. There were no statistically significant differences in mean maternal or paternal education when comparing the open repair and endoscopic repair groups. Approximately one-fourth of the participants had received a learning-related diagnosis (11 participants [24%; 95% CI, 12%-36%] in the endoscopic group and 9 participants [27%; 95% CI, 12%-42%] in the open repair group). Children in the open repair group were approximately twice as likely as those in the endoscopic group to receive physical and/or occupational therapy, mental health treatment, speech or language therapy, and/or a special education designation. A breakdown of participant characteristics by site is found in eTable 1 in Supplement 1.

**Table 2.** Participant and Familial Characteristics of the Endoscopic Repair and Open Repair Cohorts

Characteristic	Participants by procedure, No. (%) (N = 81)		*P* Value
	Endoscopic repair (n = 46)	Open repair (n = 35)	
Highest maternal education			
Partial or less than high school	2 (4)	2 (6)	.17
High school graduate	5 (11)	2 (6)	
Partial college, at least 1 year of specializing	7 (15)	13 (37)	
Standard college or university graduation	19 (41)	11 (31)	
Graduate or professional training	13 (29)	6 (17)	
Unknown	0	1 (3)	
Highest paternal education			
Partial or less than high school	3 (7)	3 (8)	.85
High school graduate	7 (15)	8 (23)	
Partial college, at least 1 year of specializing	8 (17)	7 (20)	
Standard college or university graduation	12 (26)	8 (23)	
Graduate or professional training	13 (28)	6 (18)	
Unknown	3 (7)	3 (8)	
Child education			
Home-school	4 (9)	2 (6)	.05
Public	27 (59)	29 (80)	
Private	5 (11)	4 (11)	
Unknown	10 (21)	1 (3)	
Learning-related diagnoses			
Learning disorder	3 (7)	3 (9)	.88
Language disorder	4 (9)	1 (3)	
Sensory integration disorder	1 (2)	1 (3)	
Attention deficit disorder, attention-deficit/hyperactivity disorder, or behavioral disorder	2 (4)	2 (6)	
Depression or anxiety	1 (2)	2 (6)	
Services received			
Physical or occupational therapy	3 (7)	5 (14)	<.001
Hearing services	0	1 (3)	
Speech or language therapy	8 (17)	15 (43)	
Mental health treatment	3 (7)	5 (14)	
School special education designation	2 (4)	5 (14)	
Neuropsychological testing	4 (9)	3 (9)	

### Neurodevelopment Outcome Measures

Mean DAS-II composite outcome scores for the endoscopic, open repair, and control groups were within the average range as compared with test norms in both unadjusted and adjusted analyses (Table 3). Prior to adjusting for covariates, children who had undergone endoscopic repair or open repair demonstrated no statistically significant or clinically meaningful differences in any of the DAS-II composite outcomes. As compared with the unaffected controls, participants within the open repair cohort scored significantly lower in verbal ability (control unadjusted mean score, 107; 95% CI, 105-110 vs open repair unadjusted mean score, 99; 95% CI, 95-104; mean difference, 8 points; *P* = .02). Participants within the endoscopic cohort also scored significantly lower in working memory than the unaffected controls (control unadjusted mean score, 104; 95% CI, 102-106 vs endoscopic repair unadjusted mean score, 97; 95% CI, 93-101; mean difference, 7 points; *P* = .008).

**Table 3.** Comparison of Mean Intelligence Quotient and Achievement Scores Among the Unaffected Controls, Endoscopic Repair, and Open Repair Cohorts

DAS-II subtest	Unadjusted score, mean (95%CI)			Difference between repair groups (95% CI)^b^	*P* value^c^	Adjusted score, mean (95%CI) ^a^			Difference between repair groups (95% CI)^b^	*P* value^c^
	Control	Endoscopic repair	Open repair			Control	Endoscopic repair	Open repair		
General conceptual ability	107 (104 to 109)	102 (98 to 106)	101 (97 to 106)	1 (−7 to 8)	>.99	107 (105 to 109)	100 (96 to 104)	103 (98 to 108)	−3 (−11 to 5)	>.99
Verbal ability	107 (105 to 110)	102 (98 to 107)	99 (95 to 104)	3 (−5 to 11)	>.99	107 (105 to 110)	101 (96 to 105)	101 (96 to 106)	0 (−9 to 8)	>.99
Nonverbal reasoning	104 (102 to 107)	101 (96 to 105)	99 (94 to 104)	2 (−7 to 10)	>.99	104 (102 to 107)	99 (94 to 103)	99 (94 to 105)	0 (−10 to 8)	>.99
Working memory	104 (102 to 106)	97 (93 to 101)	100 (95 to 104)	−3 (−10 to 5)	>.99	104 (102 to 106)	98 (93 to 102)	101 (97 to 106)	−3 (−12 to 4)	.83
Processing speed	102 (99 to 104)	105 (101 to 109)	101 (96 to 106)	4 (−3 to 12)	.54	102 (100 to 104)	103 (99 to 108)	102 (96 to 107)	1 (−7 to 11)	>.99

Abbreviation: DAS-II, Differential Ability Scales-II.

^a^ Adjusted for age at DAS-II assessment, sex, and socioeconomic status.

^b^ Calculated as endoscopic repair − open repair.

^c^ Pairwise comparisons corrected with the Bonferroni method.

After adjusting for covariates (age at DAS-II assessment, sex, and SES), children who had undergone endoscopic or open repair exhibited no statistically or clinically meaningful differences in the DAS-II General Conceptual Ability score (endoscopic repair adjusted mean score, 100; 95% CI, 96-104 vs open repair adjusted score, 103; 95% CI, 98-108; *P* > .99). There were also no statistically significant or clinically meaningful differences in any of the DAS-II composite subscale scores between the 2 repair groups. As compared with the unaffected controls, participants who underwent endoscopic repair scored lower on general conceptual ability (control adjusted mean score, 107; 95% CI, 105-109 vs endoscopic repair adjusted mean score, 100; 95% CI, 96-104; mean difference, 7 points; *P* = .02) and working memory (control adjusted mean score, 104; 95% CI, 102-106 vs endoscopic repair adjusted mean score, 98; 95% CI, 93-103; mean difference, 6 points; *P* = .02). However, all scores were within the normal range. A breakdown of cognitive outcomes by center can be found in eTable 2 in Supplement 1.

### Self-Reported Cognitive Function Outcome Measure

**Figure 2.**
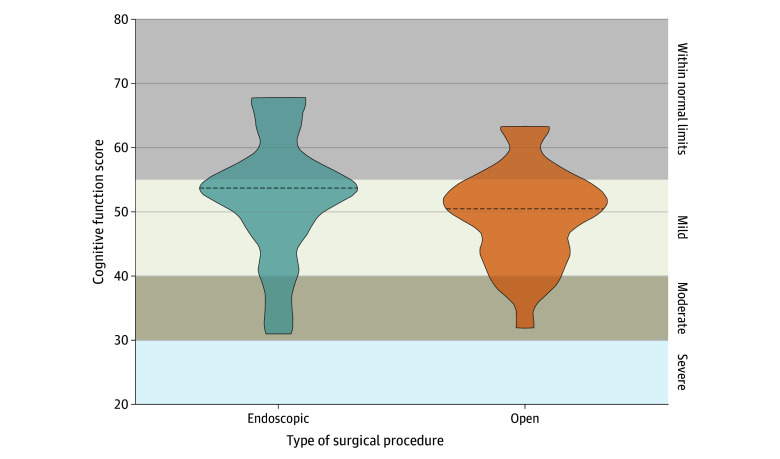
Cognitive Function as Measured by Patient-Reported Outcome Questionnaires The figure shows cognitive function scores from the Patient-Reported Outcomes Measurement Information System Cognitive Function Questionnaire for participants who underwent endoscopic and open surgery. Dotted lines indicate the median scores.

Median (range) patient or parent-reported cognitive function scores were 54 (31-68) for endoscopic repair and 50 (32-63) for open repair (P = .14) (Figure 2). We found no significant difference in scores based upon repair technique, with most patients scoring within the normal or mild patient-perceived cognitive deficit range.

## Discussion

In this cohort study, we found no statistically significant differences in DAS-II general conceptual ability scores at school-age between patients who had been treated with endoscopic or open surgery. In addition, mean general conceptual ability scores were within 1 SD of the norm for all 3 groups, both before and after controlling for relevant covariates. When comparing the treatment groups with the unaffected controls, differences in DAS-II subscale scores for verbal ability and working memory were observed. Before adjusting for covariates, patients who underwent open repair had lower verbal ability scores compared with unaffected controls, but this difference did not hold after adjusting for age at testing, sex, and SES. For the working memory subscale, patients in the endoscopic group scored lower than those in the control group, and this difference remained after controlling for covariates.

This study outcome appears to differ from one of the earliest studies^21^ to compare cognitive outcomes for children based on repair technique used. Hashim et al^21^ assessed the association of 2 different open surgical techniques with cognitive outcomes. They reported better cognitive outcomes after surgical repair using a whole vault cranioplasty technique compared with strip craniectomy. However, their patients who underwent strip craniectomy were treated with an open modified pi technique rather than a minimally invasive strip craniectomy procedure, and postoperative helmeting was not done. Additionally, the mean intelligence quotient scores for patients in the whole vault group were unusually high compared with test norms (1.5 SD higher than the expected mean), suggesting the presence of high-scoring outliers who skewed the group mean.^21^

A common concern among craniofacial surgeons is that patients with lower SES may not have access to minimally invasive surgical options. Indeed, prior research^40,41^ suggests that this may be the case, with patients of lower SES more likely to be seen and treated at older ages compared with patients of higher SES. We identified a significant difference between the endoscopic repair and open repair groups when comparing Hollingshead categories. One explanation for this trend is that children of lower SES with craniosynostosis are not referred for surgery at an early enough age for endoscopic surgery. Another explanation is that children of lower SES may not have the means to travel to centers that perform endoscopic repair or have access to postoperative helmet orthotic therapy. Educating primary care clinicians, especially those who serve children of lower SES, about different options for craniosynostosis surgery is crucial to ensure early referral of these patients so that all treatment options are still viable.

The level of parental educational attainment is known to be associated with child cognitive functioning in middle childhood,^42^ so we compared both maternal education and paternal education for the endoscopic repair vs open surgical repair group to ensure that any observed differences in cognitive functioning between groups could not be attributed to parental education. There was no statistically significant difference between groups based on parental education.

Our results suggest that patients in both the endoscopic repair and open repair groups received services at a higher rate than would be expected in the general population (15%).^43^ This finding is similar to that reported by Peck et al.^44^ As in that earlier study,^44^ patients in our study were particularly more likely to receive speech and language therapy than is typically seen in the US. This finding points to a less-studied finding, namely, that patients with a history of craniosynostosis may be more likely than their unaffected peers to require support services well into childhood.

### Limitations

This study has limitations. Despite this study including 3 centers providing cognitive outcome follow-up into adolescence, the power to find differences between repair techniques was limited by the number of patients enrolled. In the open repair group, different techniques were considered, including total cranial vault remodeling and modified pi procedures. Because multiple surgeons guided treatment within individual institutions across the study time course, these technical differences occurred both between and within institutions. Due to the cohort sizes, we were not able to assess center or surgeon effects; this may make the study more generalizable in a clinical setting but prevents comparisons between different open repair techniques or between a specific open repair and endoscopic repair procedure. That said, in an a priori power analysis, we estimated 80% power to detect differences greater than 5 points on our standardized measures (ie, 0.3 SD). This would be a modest, but potentially clinically meaningful difference; none of our observed differences crossed this threshold.

Another potential limitation to this study is selection bias. Of 256 patients identified as eligible for inclusion, 175 (46%) were excluded either because it was not possible to reach the family or the family declined to participate. It is theoretically possible that families systematically excluded themselves based on the patient’s neurodevelopmental status. We did not have any indication that this was the case and attributed the high failure rate to reach families to the fact that it had been many years since the original surgical corrections were done. However, we cannot eliminate the possibility that patients disproportionately declined to participate based on developmental status.

Despite these limitations, this study adds to the limited literature addressing the study questions. Larger scale noninferiority studies and/or systematic reviews may more fully answer these questions. Randomized clinical trials would, of course, be the benchmark but are unlikely without observational studies like this study to establish clinical equipoise. In addition, it would be almost impossible to have a true randomized clinical trial given that it would be very difficult to have parents give up the ability to choose their child’s type of surgery.

Future research should take into account potential genetic confounders. It has been suggested that genetic influences may play a role in the neurodevelopment of patients with nonsyndromic craniosynostosis; brain development might be compromised in individuals with craniosynostosis independent of the effect that premature skull fusion might have had on brain expansion and development.^45,46^

## Conclusions

In this cohort study of 81 school-age patients who had undergone repair of nonsyndromic craniosynostosis at a young age (46 who underwent endoscopic repair and 35 who underwent open repair), we found no association of procedure type (endoscopic vs open) with cognitive outcomes. This study can be used as a basis for a larger study. This information is needed for families and surgeons to make informed treatment decisions. Early referral by pediatricians will allow clinicians to offer both open calvarial vault surgery and minimally invasive options to families.

## Notes

Supplement 1. **eTable 1.** Demographics and Participant Characteristics of the Endoscopic and Open Repair Cohorts by Center **eTable 2.** Comparison of Unadjusted Achievement Scores Between Centers in the Endoscopic and Open Repair Cohorts

Supplement 2.
Data Sharing Statement
