# Supplementary material for: Cognitive Outcomes of Children With Sagittal Craniosynostosis Treated With Either Endoscopic or Open Calvarial Vault Surgery
Source: JAMA Netw Open. 2024 Apr 29;7(4):e248762. doi: 10.1001/jamanetworkopen.2024.8762 (PMC11059043; doi:10.1001/jamanetworkopen.2024.8762)
Supplement: Supplement 1. — eTable 1. Demographics and Participant Characteristics of the Endoscopic and Open Repair Cohorts by Center eTable 2. Comparison of Unadjusted Achievement Scores Between Centers in the Endoscopic and Open Repair Cohorts [file jamanetwopen-e248762-s001.pdf]

Supplemental Online Content

Magge SN, Fotouhi AR, Allhusen V, et al. Cognitive outcomes and sagittal craniosynostosis treated with endoscopic surgery or open calvarial vault remodeling. *JAMA Netw Open*. 2024;7(4):e248762. doi:10.1001/jamanetworkopen.2024.8762

eTable 1. Demographics and Participant Characteristics of the Endoscopic and Open Repair Cohorts by Center

eTable 2. Comparison of Unadjusted Achievement Scores Between Centers in the Endoscopic and Open Repair Cohorts

This supplemental material has been provided by the authors to give readers additional information about their work.

© 2024 Magge SN et al. *JAMA Network Open.*

eTable 1. Demographics and Participant Characteristics of the Endoscopic and Open Repair Cohorts by Center

		Endoscopic			Open			
		**Center 1**	**Center 2**	***p***	**Center 1**	**Center 2**	**Center 3**	***p***
		**(N = 25)**	**(N = 21)**		**(N = 19)**	**(N = 11)**	(n = 5)	
**Median Age at Initial Presentation, Months (Range)**		2 (0-6)	2 (0-6)	*0.930*	10 (1-32)	3 (0-9)	3 (3-9)	***< 0.001***
**Median Age at DAS-II Testing, Years (Range)**		8 (6-11)	7 (5-10)	***0.015***	8 (5-14)	8 (6-14)	8 (7-9)	*0.882*
**Sex, frequency (%)**				*0.325*				***< 0.001***
	Female	9 (36)	4 (19)		5 (26)	4 (36)	0 (0)	
	Male	16 (64)	17 (81)		14 (74)	7 (64)	5 (100)	
**Race, frequency (%)**				***< 0.001***				***< 0.001***
	African American	0 (0)	4 (19)		2 (11)	0 (0)	4 (80)	
	Asian	0 (0)	0 (0)		0 (0)	2 (18)	0 (0)	
	White	25 (100)	11 (52)		17 (89)	6 (55)	0 (0)	
	More Than One Race	0 (0)	4 (19)		0 (0)	0 (0)	0 (0)	
	Unknown	0 (0)	2 (10)		0 (0)	3 (27)	1 (20)	
**Ethnicity, frequency (%)**				*0.545*				*0.437*
	Hispanic or Latino	0 (0)	3 (11)		1 (6)	2 (18	1 (20)	
	Not Hispanic or Latino	25 (100)	18 (89)		18 (94)	9 (82)	4 (80)	
**Gestation, frequency (%)**				*0.239*				*1.000*
	^{3} *37 Weeks*	22 (85)	21 (100)		17 (88)	10 (91)	5 (100)	
	< 37 Weeks	3 (15)	0 (0)		2 (12)	1 (9)	0 (0)	
**Mean Hollingshead Total Score (SD)**		28 (11)	49 (15)	*0.83*	43 (14)	45 (18)	41 (12)	*0.883*

statistically significant differences between centers ($p < 0.05$) are in **bold.**

© 2024 Magge SN et al. *JAMA Network Open.*

eTable 2. Comparison of Unadjusted Achievement Scores Between Centers in the Endoscopic and Open Repair Cohorts

	Endoscopic			Open			
	**Center 1**	**Center 2**	***p***	**Center 1**	**Center 2**	**Center 3**	***p***
**DAS-II Composite** Mean (95% CI)	102 (70 – 134)	105 (79 – 131)	*0.369*	97 (66 – 129)	113 (77 – 150)	96 (86 – 110)	***0.032***
**Global Cognitive Ability**	102 (70 – 134)	103 (76 – 130)	*0.904*	98 (66 – 130)	111 (74 – 149)	93 (66 – 121)	*0.068*
**Verbal Ability**	104 (76 - 135)	101 (73 – 133)	*0.248*	99 (67 – 131)	104 (77 – 131)	91 (42 – 139)	*0.361*
**Nonverbal Reasoning**	99 (67 – 132)	102 (80 - 124	*0.440*	96 (56 – 135)	107 (74 – 141)	94 (80 – 107)	*0.198*
**Working Memory**	97 (62 – 133)	97 (65 – 129)	*0.998*	98 (72 – 124)	105 (88 – 121_	95 (69 – 122)	*0.228*
**Processing Speed**	99 (64 – 134)	114 (80 – 147)	***0.007***	98 (70 – 126)	109 (88 – 130)	94 (81 – 106)	***0.038***

© 2024 Magge SN et al. *JAMA Network Open.*
