# Supplementary material for: Cognitive Outcomes of Children With Sagittal Craniosynostosis Treated With Either Endoscopic or Open Calvarial Vault Surgery
Source: JAMA Netw Open. 2024 Apr 29;7(4):e248762. doi: 10.1001/jamanetworkopen.2024.8762 (PMC11059043; doi:10.1001/jamanetworkopen.2024.8762)
Supplement: Supplement 2. — Data Sharing Statement [file jamanetwopen-e248762-s002.pdf]

Data Sharing Statement

Magge. Cognitive Outcomes of Children With Sagittal Craniosynostosis Treated With Either Endoscopic or Open Calvarial Vault Surgery. *JAMA Netw Open*. Published April 29, 2024. doi:10.1001/jamanetworkopen.2024.8762

Data
Data available: No

Additional Information
Explanation for why data not available: Could share deidentified data if necessary and it complies with all IRB's.
